# Silencing of XRCC4 increases radiosensitivity of triple-negative breast cancer cells

**DOI:** 10.1042/BSR20180893

**Published:** 2019-03-19

**Authors:** Yuqing Wen, Gongpeng Dai, Liping Wang, Kanda Fu, Shuguang Zuo

**Affiliations:** 1 Department of General Surgery, Huaihe Hospital of Henan University, Kaifeng, Henan Province 475001, China; 2 Center for Translational Medicine, Huaihe Hospital of Henan University, Kaifeng, Henan Province 475001, China; 3 Institute of Infection and Immunity, Huaihe Hospital of Henan University, Kaifeng, Henan Province 475001, China

**Keywords:** progression-free survival, radiosensitivity, RNA interference, triple negative breast cancer, X-ray repaircross complementing protein 4

## Abstract

Background: Radiotherapy is an important locoregional treatment, and its effect on triple-negative breast cancer (TNBC) needs to be enhanced. The aim of the present study was to investigate the potential effects of XRCC4 on radiosensitivity of TNBC. Methods: The RNAi technique was implemented to establish the TNBC stable cell line with XRCC4 knockdown. MTT assay was used to detect the effect of XRCC4 knockdown on cell proliferation. Western blot and immunohistochemistry assays were employed to identify protein expression. Colony assay was performed to detect the effect of XRCC4 knockdown on the colony formation ability of TNBC cells with radiation treatment. Comet assay was conducted to evaluate the influence of XRCC4 silencing on DNA repair activity in ionizing radiation. In addition, we performed a survival analysis based on data in TCGA database. Results: XRCC4 knockdown by lentivirus-mediated shRNA had no significant effect on proliferation of TNBC cells. Knockdown of XRCC4 could substantially increase the sensitivity of TNBC cells to ionizing radiation. The DNA damage level was detected to be increased in the XRCC4 knockdown group, indicating there was a significant repair delay in the XRCC4-deleted cells. Clinical sample analysis exhibited that there were various XRCC4 expression in different patients with TNBC. Moreover, survival analysis showed that high expression of XRCC4 was significantly associated with poor progression-free survival after radiotherapy in TNBC patients. Conclusion**:** Our findings suggest that XRCC4 knockdown sensitizes TNBC cells to ionizing radiation, and could be considered as a novel predictor of radiosensitivity and a promising target for TNBC.

## Introduction

With 2.4 million incident cases and 523,000 deaths per year, breast cancer is the most commonly diagnosed malignancy and the leading cause of cancer deaths among women [1]. In Chinese women, breast cancer is one of the most extensively increasing cancers because of socioeconomic changes and unique reproductive patterns [2,3]. Triple-negative breast cancer (TNBC) is an aggressive subtype of breast cancer, accounting for approximately 15% of all cases [4]. It is characterized by the absence of estrogen receptors, progesterone receptors and human epidermal growth factor receptor 2, thereby making it refractory to current traditional endocrine therapy and targeted therapy [4,5]. Relative to other phenotypes of breast cancer, TNBC has an increased likelihood of recurrence, distant metastasis and mortality. Current treatment involves surgery, chemotherapy and radiotherapy. As an integral part of cancer management, radiation therapy is an important locoregional treatment modality, contributing to more than 50% of cancer treatment [6,7]. However, the efficacy of radiotherapy is limited by its own strong side effects that are usually dose dependent [8,9]. Due to the heterogeneity of TNBC, cells present various sensitivities to radiation. Some cancer cells may be intrinsically resistant to ionizing radiation induced damages, resulting in poor tumor local control. Therefore, it is necessary to explore promising markers for predicting or improving the radiosensitivities of TNBC.

It is well known that ionizing radiation leads to cell death by inducing DNA double-strand breaks (DSBs). Therefore, inhibiting DSBs repair is an efficient way to sensitize cancer cells to ionizing radiation-induced killing. In mammal, cells achieve DNA DSBs repair through non-homologous end joining (NHEJ) and homologous recombination (HR). X-ray repair cross-complementing gene 4 (XRCC4) is a core member in NHEJ pathway, which forms a complex with DNA ligase IV to repair DNA DSBs [10,11]. It has been demonstrated that RNAi-mediated depletion of XRCC4 can successfully manipulate the NHEJ pathway [12]. Combining amiR and siRNA, XRCC4 could efficiently increase the radiosensitization of glioblastoma multiforme and lung cancer cells [13]. Hori et al. [14] reported that low expression of XRCC4 could prolong the survival time of esophageal cancer patients, suggesting that low expression of XRCC4 might have a relationship with the increase of radiosensitivity. XRCC4 polymorphisms have also been linked to a risk of susceptibility for breast cancer [15]. These findings suggest that XRCC4 is closely associated with the radiosensitivity of various cancers.

However, few studies investigated the significance of XRCC4 gene expression on the radiosensitivity of TNBC. We hypothesize that the expression level of XRCC4 gene will affect the radiosensitivity of TNBC cells and influence the survival time of TNBC patients who underwent radiotherapy. In the present study, TNBC stable cell line with XRCC4 gene knockdown was established by RNA inference to investigate the radiosensitizing effect of XRCC4 knockdown on TNBC cells. Meanwhile, the expression value of XRCC4 was detected in clinic. In addition, we investigated the impact of XRCC4 expression on patient survival in TNBC who underwent radiotherapy. Our study will be of great emphasis for future prevention and control strategy of TNBC.

## Methods

### Cell culture

Human breast cancer cell line MDA-MB-231, one of the most commonly studied cell lines of TNBC, was obtained from School of Basic Medical Sciences, Fourth Military Medical University, and were cultured in Dulbecco’s modified eagle medium (DMEM, Hyclone, Logan, U.S.A.) supplemented with 10% fetal bovine serum (FBS), at 37°C with 5% CO_2_ humidified atmosphere. The triple-negative nature of the cell line was tested by Immunohistochemistry test. HEK-293T cells were from our laboratory and maintained in DMEM supplemented with 10% FBS, 100 μg/ml streptomycin and 100 U/ml penicillin.

### Tumor samples

The present study considered 32 TNBC patients who had undergone a resection of primary tumors at Huaihe Hospital of Henan University between January 2011 and March 2016. All of the patients were female, with age ranging from 27 to 68 years old. Tumor stage (TNM) was evaluated according to the American Joint Committee on Cancer staging system. The patient proportion of each tumor status for T_I_, T_II_ and T_III_ was 10, 17 and 5, respectively. All patients were histologically confirmed as TNBC. The procedure was reviewed and approved by the ethics committee of Henan University. Human tissue acquisition and usage in the present study complied with the National Regulations on the Use of Clinical Samples in China. The research has been carried out in accordance with the World Medical Association Declaration of Helsinki, and written informed consent for participation in the study was obtained from all participants. Adjacent normal breast tissue was obtained for negative control.

### XRCC4 shRNA construction

In the present study, short hairpin (sh)RNA was used to silence XRCC4 expression in MDA-MB-231 cells. In the present study, shRNA sequences for XRCC4 were as follows: (forward) 5′-CACCGCCTCAGGAGAATCAGCTTCAACTCGAGTTGAAGCTGATTCTCCTGAGGTTTTTT-3′ and (reverse) 5′- AAACAAAAAACCTCAGGAGAATCAGCTTCAACTCGAGTTGAAGCTGATTCTCCTGAGGC-3′. As negative controls, the cells were transducted or transfected with non-targeting shRNAs. We first constructed the lentiviral expression vector pLenti-U6-EF1a-copGFP-P2A-Puro, which carried both green fluorescent protein (GFP) and puromycin gene, based on vectors lentiCRISPR v2 (Addgene, Cambridge, MA, U.S.A.) and pCDH-copGFP-Puro (Systembio, Palo Alto, CA, U.S.A.). Then, XRCC4 shRNA oligos were cloned into above vector to construct XRCC4 shRNA expression lentiviral vector pLenti-U6-XRCC4-EF1a-copGFP-P2A-Puro. The XRCC4 shRNA lentivirus constituted a pool of concentrated, transduction-ready viral particles containing XRCC4-specific constructs. XRCC4 shRNA expression vector were confirmed by direct DNA sequencing (Sangon Biotech, Shanghai, China).

### Lentiviral packaging and infection

Human breast cancer cell line MDA-MB-231 was used for stable infection with XRCC4-targeting shRNA lentivirus particles. Briefly, HEK-293T cells were seeded with approximately 4 × 10^5^ per well in 12-well plates and incubated overnight. The XRCC4 shRNA (or empty) expression vector pLenti-U6-XRCC4-EF1a-copGFP-P2A-Puro with the packaging plasmids pMD2.G (Addgene) and psPAX2 (Addgene) were formed into a complex with jetPEI transfection reagent (Polyplus-transfection, Illkirch, France) and co-transfected into the HEK-293T cells, according to the manual. To infect MDA-MB-231 cells, the pooled and clarified supernatants from transfected HEK 293T cells were centrifuged to concentrate the viruses. These viruses were titered for infecting MDA-MB-231 cells in the presence of polybrene. The transduced cells were harvested after 72-h postinfection for further experiments. Cells were treated with 5 μg/ml puromycin to select stable cells with XRCC4 knockdown. Transduction efficiency was measured using fluorescence microscopy based on the percentage of GFP-positive cells.

### Western blot

Western blot was implemented for quantification of protein expression. Cells were washed twice with ice-cold phosphate-buffered saline (PBS) and were homogenized with radioimmunoprecipitation assay lysis buffer (Cwbiotech, Bejing, China) containing protease inhibitor. Protein concentration was determined by BCA assay (Cwbiotech, Beijing, China). For Western blot, 40 ng of protein extracts were electrophoresed with 10% sodium dodecyl sulfate polyacrylamide gels, then were transferred to nitrocellulose membrane (Pall Laboratory, NewYork, NY, U.S.A.). The membrane was blocked with 5% non-fat milk at room temperature for 2 h, then incubated with rabbit anti-XRCC4 primary antibody (1:1000, Proteintech, Rosemont, IL, U.S.A.) and anti-GAPDH antibody (1:10,000, Proteintech) at 4°C overnight. The membrane was washed with Tris-buffered saline with 0.1% Tween-20 three times, then was incubated with goat anti-rabbit IgG conjugated horseradish peroxidase secondary antibody (1:5000, Proteintech) at room temperature for 1 h. The signals were detected using Western Lightning Plus ECL (Proteintech) and analyzed by ChemiDoc XRS (Bio-Rad, U.S.A.).

### Immunohistochemistry test

Tumor cryosections or cells grown on chamber slides were fixed in 4% paraformaldehyde for 20 min at room temperature, and specimens were blocked with blocking serum at 37°C for 20 min. Then specimens were incubated with rabbit anti-human primary antibody (1:200, Proteintech) at 37°C for 2 h, and washed three times with PBS, followed by incubation with HRP-conjugated goat anti-rabbit secondary antibody (1:200, Proteintech) for 30 min at 37°C. Immunostaining was visualized using 3,3′-diaminobenzidine reagent followed by counterstaining with hematoxylin. Omitting primary antibody was considered as a negative control.

### MTT assay

The effect of XRCC4 knockdown on the proliferation of breast cancer cells was tested by MTT (3-[4,5-dimethylthiazol-2-yl]-2,5-diphenyltetrazolium bromide) assay [16]. In brief, untransducted cells, empty vector-transducted cells and shRNA-transducted cells were respectively seeded with 4000 per well in 96-well plates and incubated overnight. After washing twice with PBS, cells were incubated in the presence of 20 μl MTT for an additional 4 h at 37°C. Then, the medium was removed and replaced with 150 µl dimethylsulfoxide to solubilize MTT formazan at 37°C for 10 min. The values of optical density of the solutions were measured by spectrophotometry (D30, Eppendorf, Hamburg, Germany) at a 490-nm test wavelength.

### Colony formation assay

Colony assay was performed to test the clonogenic survival of stable transducted cells. An appropriate number of cells (200 per well) were plated into 12-well dishes with fresh growth medium at 37°C with 5% CO_2_. After 24 h, breast cancer cells were exposed to X-rays with a single dose of 0, 2, 4 and 6 Gy, emitted from an irradiator (Precise VMAT, Elekta, Stockholm, Sweden). After 16 days, when cell colonies were formed, the cells were washed with PBS and fixed using 3.7% paraformaldehyde for 15 min, and then stained with 0.1% Crystal Violet solution for 15 min at room temperature. The visible colonies with more than 50 cells were counted using ChemiDoc XRS image screening system (Bio-Rad). In each irradiation dose group, surviving fraction of cells was calculated as the plating efficiency of the irradiation cells divided by that of the non-irradiated control.

### Comet assay

In order to determine the effect of XRCC4 knockdown on the repair of ionizing radiation-induced DNA damage, we conducted comet assay. In detail, the untransduced, vector transduced and XRCC4 shRNA transduced MDA-MB-231 cells were subjected to 4 Gy irradiation and subsequently incubated in normal medium for 15 and 60 min before performing the Comet assay. DNA damage was evaluated using the alkaline comet assay, according to the manufacturer's instructions (Trevigen, Gaithersburg, MD, U.S.A.). The tail moment (% DNA in tail × tail length) was determined for 50 cells using the CometScore software (TriTek, Sumerduck, VA, U.S.A.).

### Survival analysis

An amount of preliminary RNASeq data involved in breast cancer has been reported in TCGA (www.cancaergenome.nih.gov), which have provided useful insights to derive the prognostic and predictive data. In the present study, a total of 1222 individuals with breast cancer were obtained from the TCGA data portal (https://portal.gdc.cancer.gov/). Removing patients with non-triple-negative breast cancer (nTNBC, *n*=760) and patients with undefined Her-2 status (*n* = 308) from the study, there were only 154 individuals with TNBC. Among them, only 20 patients who received radiotherapy (with mean radiation dose of 34 Gy) and contained complete follow-up information were remained for progression-free survival (PFS) analysis. There were 17 TNBC patients who experienced a complete response to radiotherapy and 3 patients with progressive disease after radiotherapy. The characteristics of these patients were recorded in Table 1. Based on the expression of XRCC4 in these 20 TNBC patients, expression level greater than the median was classified as high expression; otherwise it was classified as low expression. The PFS was calculated in days from surgery to cancer progression or causing death.

**Table 1:** Clinical characteristics of XRCC4^low^ and XRCC4^high^ patients

Variables	XRCC4^low^, *N* = 10	XRCC4^high^, *N* = 10	χ^2^	*P*
Age				
<60	6	8	0.905	0.342
>60	4	2		
Pathologic_stage				
I-II	8	8	0	1
III-IV	2	2		
Stage_T				
T1-T2	9	8	0.373	0.542
T3-T4	1	2		
Stage_N				
N0	6	6	0	1
NX	4	4		
Stage_M				
M0	6	9	2.280	0.131
MX	4	1		
Progression				
NO	10	7	3.353	0.067
YES	0	3		

### Statistical analysis

Each experiment was repeated at least three times. Statistical analyses were executed using SPSS20.0 software (SPSS Inc., Armonk, NY, U.S.A.). Continuous variables were expressed as mean ± standard deviation, while categorical variables were reported as frequencies (%). To analyze the data, Chi-square test, one-way ANOVA and LSD test were used. *P*-value <0.05 was defined as significant level.

## Results

### Lentivirus-mediated shRNA efficiently suppresses the expression of XRCC4

In the present study, the triple-negative nature of the cell line was first confirmed by immunohistochemistry array (Supplementary Figure S1A). To investigate the effects of XRCC4 in TNBC, XRCC4 expression was knocked down in human breast cancer cell line MDA-MB-231 by lentivirus-mediated transduction. First, the lentiviral expression vectors pLenti-U6-EF1a-copGFP-P2A-Puro and pLenti-U6-XRCC4-EF1a-copGFP-P2A-Puro were constructed and used for stable infection to MDA-MB-231 cells. The transducted cells with stable expression XRCC4 shRNA or empty vector were obtained with puromycin selection. Transduction efficiency was determined using fluorescence microscopy based on the percentage of the GFP-positive cells. As shown in Supplementary Figure S1B, the expression of GFP in the MDA-MB-231 cells could be visualized, indicating that the vectors were all successfully transported into MDA-MB-231 cells. Moreover, almost all the cells expressed GFP, and the transduction efficiency was over 80%. Based on these results, MDA-MB-231 cells with stable knockdown of XRCC4 were established successfully.

**Figure 1:**
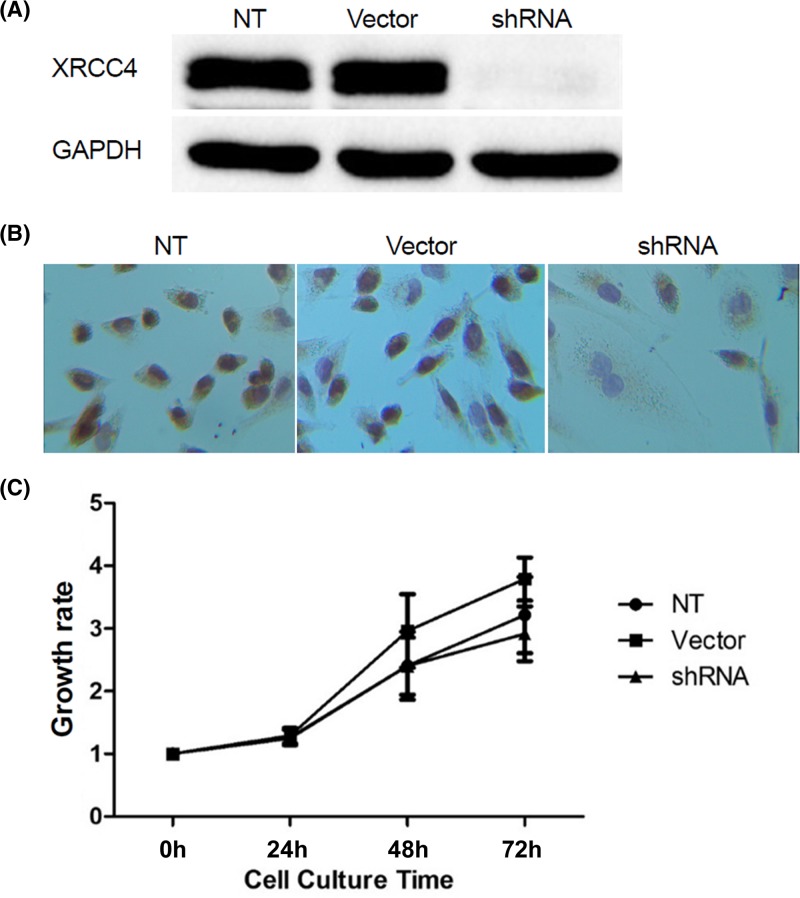
Lentivirus-mediated shRNA efficiently suppresses the expression of XRCC4 (**A**) Western blot confirms XRCC4 knockdown in MDA-MB-231 cells using lentivirus-mediated shRNA. (**B**) Immunohistochemistry test confirms XRCC4 knockdown in MDA-MB-231 cells using lentivirus-mediated shRNA. Magnification, 400×. (**C**) The effects of XRCC4 knockdown on proliferation of MDA-MB-231 cells as determined by an MTT assay. NT, untransducted control group; Vector, empty vector control group; shRNA: XRCC4 shRNA group. *P*>0.05

The quantification of XRCC4 protein levels was detected by Western blot. The results indicated that XRCC4 protein levels were highly expressed in both empty vector-transducted and untransducted cells. While XRCC4 protein levels were significantly down-regulated in XRCC4 shRNA-transducted cells compared with empty vector-transducted and untransducted cells (Figure 1A).

The immunohistochemistry test gave a consistent result, as shown in Figure 1B. In untransducted and empty vector-transducted cells, strongly positive expressions of XRCC4 were observed in almost all cells and were mainly visualized in cell nucleuses. However, most of XRCC4 shRNA-transducted cells showed negative expressions of XRCC4. We also found that a few cells presented weakly positive results may attributing to non-specific staining. These results demonstrated that lentivirus-mediated shRNA targeting XRCC4 could result in a considerable reduction of XRCC4 expression in TNBC cell line.

### XRCC4 knockdown shows no significant effect on cell proliferation

To determine the role of XRCC4 expression on cell proliferation, MTT assay was performed on MDA-MB-231 cells. As indicated in Figure 1C, there was no significant difference in cell proliferation among the untransducted, the empty vector-transducted and XRCC4 shRNA-transducted MDA-MB-231 cells at any time points (*P*>0.05).

### XRCC4 knockdown sensitizes TNBC cells to ionizing radiation

**Figure 2:**
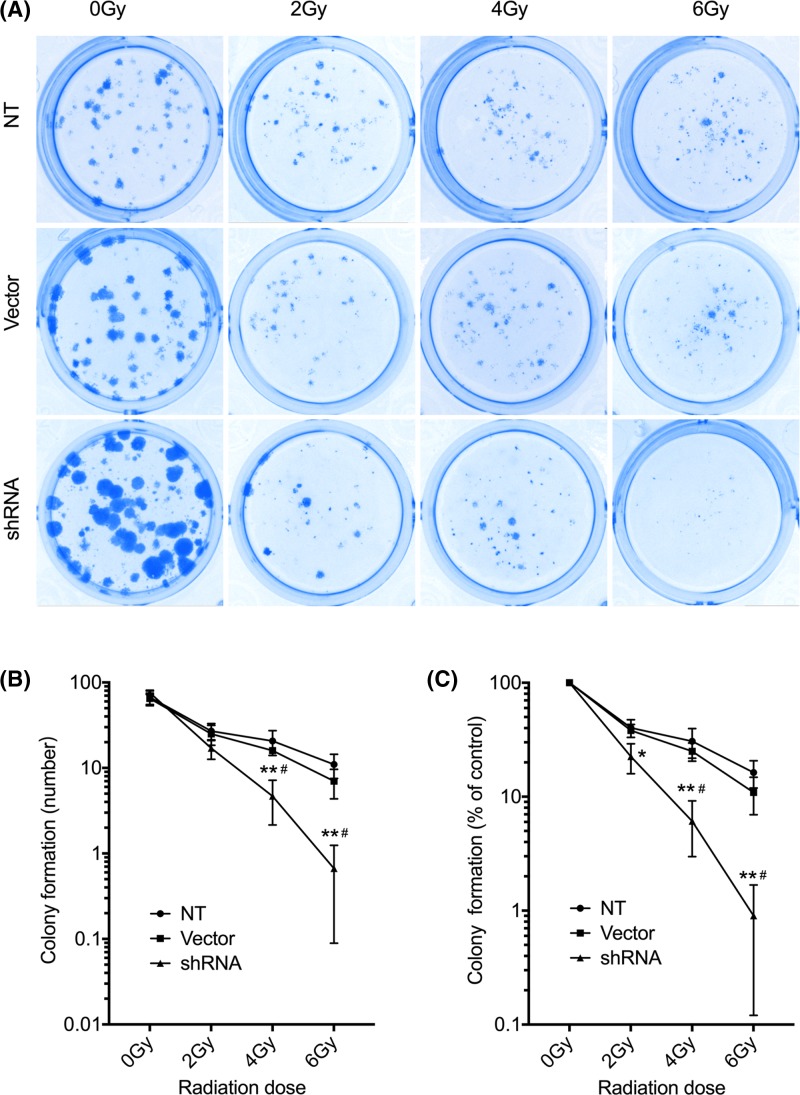
XRCC4 knockdown affects radiosensitivity of MDA-MB-231 cells (**A**) Representative images of the size and number of colonies, (**B**) quantification of the number of colonies and (**C**) quantification of the survival fraction of colonies after transducted MDA-MB-231 cells were subjected to 0, 2, 4 and 6 Gy of irradiation. NT, untransducted control group; Vector, empty vector control group; shRNA: XRCC4 shRNA group. *P<0.05 vs. NT; **P<0.01 vs. NT; ^#^*P*<0.05 vs. Vector.

The clonogenic cell survival assay was employed to determine the radiosensitivity of XRCC4 silenced cells to ionizing radiation. Our results showed that XRCC4 shRNA-transducted cells exhibited hypersensitivity to the killing effects of ionizing radiation relative to untransducted and empty vector-transducted cells (Figure 2). Representative images of the size and number of colonies per well are shown in Figure 2A. With no ionizing radiation, there was no significant difference of the number and survival rate of colony formation among three groups (*P*>0.05). From Figure 2B,C, ionizing radiation could decrease the number and survival rate of colonies in all three groups in a dose-dependent manner, and XRCC4 shRNA-transduction group decreased more gradually than the other two groups. Specifically, the colony number of XRCC4 shRNA-transducted cells was significantly lower than that of untransducted and empty vector-transducted cells with radiation dose of 4 and 6 Gy (*P*<0.05). The survival rate of colonies in XRCC4 shRNA-transduction group was significantly lower than that in untransduction and empty vector-transduction groups (*P*<0.05). While no significant difference of colony number and survival rate was found between untransducted and empty vector-transducted cells (*P*>0.05). These results indicated that XRCC knockdown can enhance the radiosensitivity of TNBC cells MDA-MB-231.

### Analysis of DNA damage based on Comet assay

**Figure 3:**
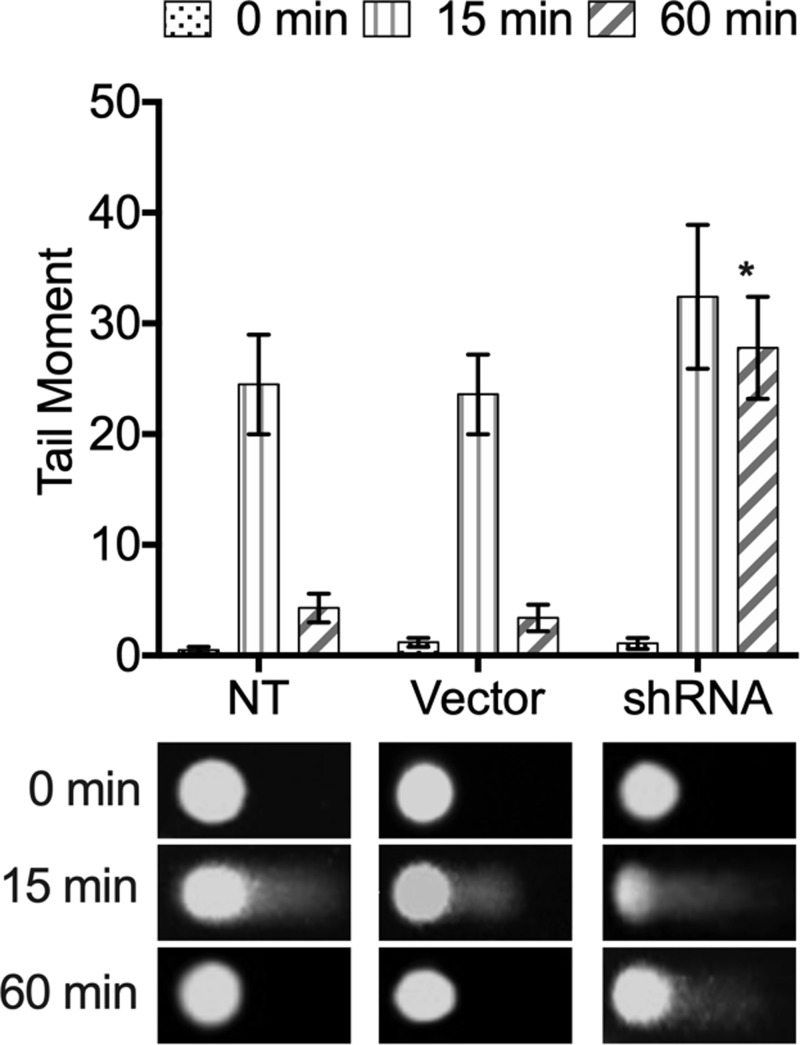
COMET assay used to evaluate the effects of XRCC4 on repair of DNA damage caused by ionizing radiation in TNBC cell line MDA-MB-231 Representative comet images after 0, 15 and 60 min following treatment were exhibited. The tail moment obtained from the comet assay was analyzed quantitatively. The level of DNA damage gradually returns to the baseline in the untransduction and empty vector-transduction groups 60 min after treatment of ionizing radiation. The DNA damage level still remains high in the XRCC4 knockdown group, indicating there is a significant repair delay in the XRCC4-deleted cells. *P<0.01 vs. NT and Vector transduced cells.

The influence of XRCC4 silencing on DNA repair activity in ionizing radiation-induced DNA damage was further assessed relying on a comet assay. The level of DNA damage gradually returned to the baseline in the untransduction and empty vector-transduction groups 60 min after treatment of ionizing radiation (*P*>0.05). However, the DNA damage level still remained high in the XRCC4 knockdown group, indicating there was a significant repair delay in the XRCC4-deleted cells (*P*<0.01, Figure 3).

### Expression of XRCC4 in TNBC tissues

**Figure 4:**
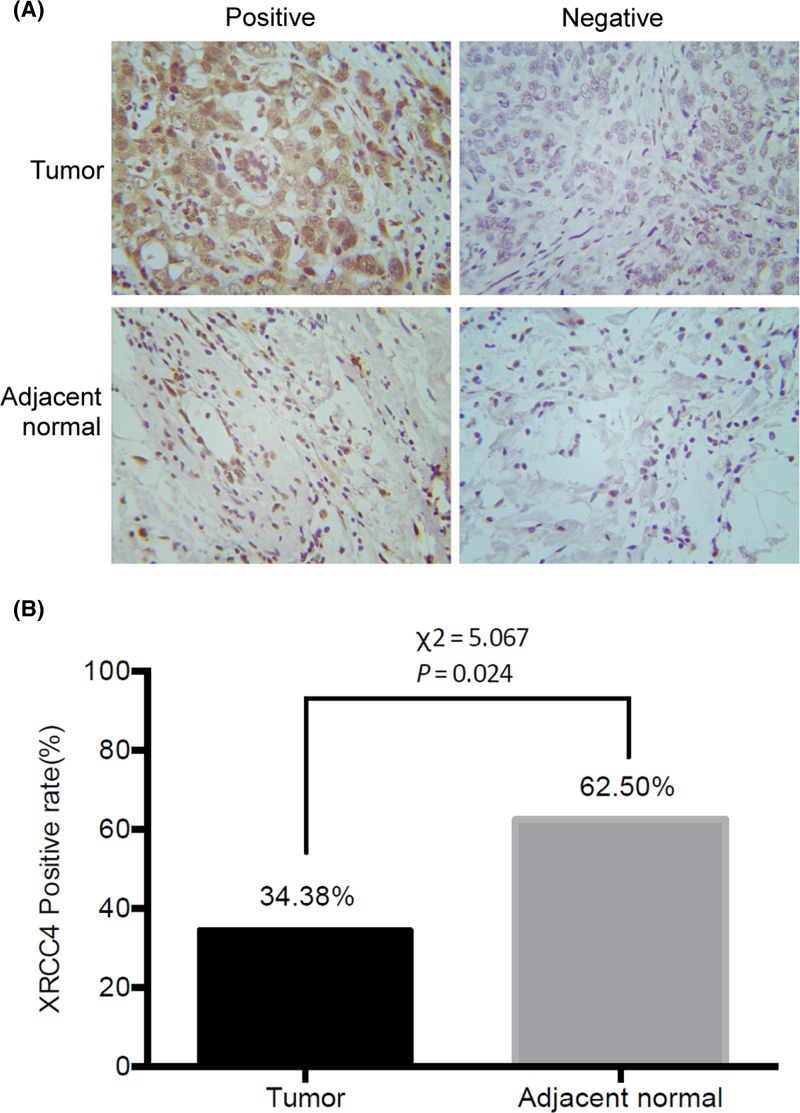
Expression of XRCC4 in TNBC tissues and adjacent normal breast tissues (**A**) Representative images of immunohistochemistry assay, and (**B**) a histogram showing XRCC4 expression positive rate in TNBC and adjacent normal breast tissues. Magnification, 200×.

In the present study, we also performed immunohistochemistry test to investigate XRCC4 expression in tissue specimens from 32 TNBC tissues and 32 adjacent normal breast tissues. Immunohistochemical analysis demonstrated that 34.38% (11/32) of TNBC samples were positive stain and 62.50% (20/32) of adjacent normal breast samples were positive stain. Representative images of immunohistochemistry assay were shown in Figure 4A. Statistical analysis indicated that XRCC4 expression decreased significantly in tumor tissues compared with adjacent normal breast tissues (Figure 4B, *P*<0.05). Additionally, there are various XRCC4 expression levels in different individuals. Given the notion of low expression of XRCC4-enhancing radiosensitivity, we could provide a customized radiation dose reduction for patients with XRCC4 low expression to avoid the unexpected side effects.

### High expression of XRCC4 involves poor PFS after radiotherapy in TNBC

**Figure 5:**
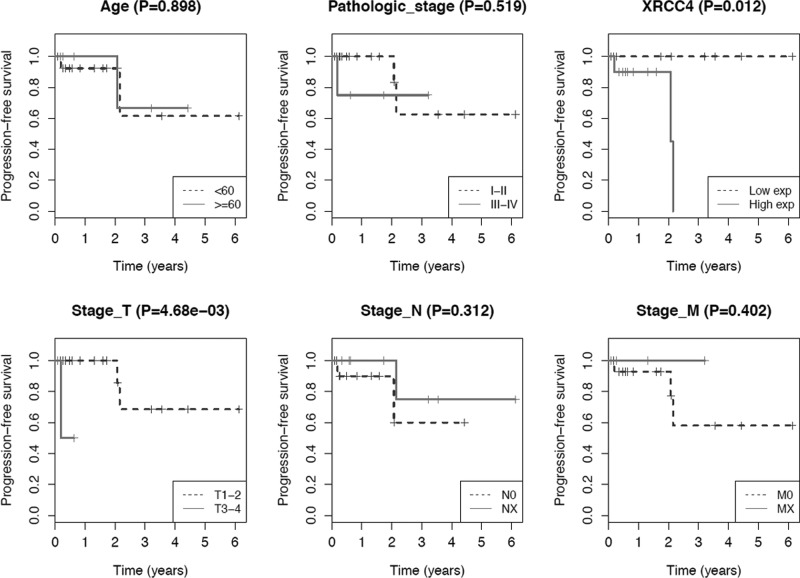
Prognostic analysis of TNBC patients who underwent radiotherapy utilizing TCGA cohort XRCC4 levels and T stage parameters are significant prognostic factors in TNBC patients after radiotherapy.

In the present study, we investigated the association of XRCC4 expression and prognosis of TNBC patients who underwent radiotherapy utilizing TCGA cohort. Patients were divided into two groups based on the mean value of XRCC4 expression levels. Out of 20 cases, 10 and 10 patients were classified as XRCC4 high- and low-expression groups, respectively. As shown in Table 1, there were no significant differences in clinical characteristics between two groups. Of 10 patients in high-expression group, 7 experienced a complete response to radiotherapy and 3 developed progressive disease after radiotherapy. While in XRCC4 low-expression group, all 10 patients experienced complete responses to radiotherapy, implying that patients with low expression of XRCC4 may more sensitive to ionizing radiation. The curves indicated that patients with high expression of XRCC4 showed a statistically significant decrease in PFS compared twith those with low expression (Figure 5, *P*=0.012). In addition, we also investigated the associations of other clinical or pathological factors and prognosis of TNBC patients with radiotherapy. The T stage parameter was a significant prognostic factor, and other clinical factors had no significant effects on PFS in TNBC patients who underwent radiotherapy (Figure 5). Our results showed that low expression of XRCC4 was associated with better PFS after radiotherapy in TNBC patients, suggesting that XRCC4 expression modulated radiosensitivity in TNBC patients and could be considered as a prognostic factor in TNBC therapy.

## Discussion

As a hard-to-treat subtype of breast cancer, TNBC poses a great challenge because of its molecular heterogeneity, aggressive nature and the lack of targeted therapies. Radiotherapy plays a crucial role in the locoregional treatment strategy of TNBC [7]. While intrinsic radioresistance and side effects can hinder efficient treatment, existing circumstances prompt the efforts to discover actionable markers to improve the radiosensitivity of TNBC.

Radiation therapy plays an important role in the management of various cancers. Ionizing radiation exposure induces DSBs of cancerous cells, resulting in cell death through the formation of chromosomal aberrations [17]. Inhibition of DSB processing determines the radiosensitivity status of cells [18]. In mammal, relative to HR pathway, NHEJ predominantly contributes to the repair of radiation-induced DSBs, which acts during any phase of the cell cycle [19,20]. NHEJ is one of the most important pathways involved in the repair of radiation-induced DNA damage. At any time in the cell cycle, DSBs can be repaired by NHEJ pathway, and not affected by cell-cycle distribution. Our MTT assay showed that XRCC4 knockdown had no significant effect on cell proliferation. NHEJ eliminates DSBs by direct ligation of the broken ends in the absence of a homologous DNA template [21]. Increasing evidence have verified that the ability of DSBs repair influences cancer susceptibility and the radiosensitivity of tumors by focusing on the activity of NHEJ [22–24]. Patients lacking normal NHEJ are sensitive to ionizing radiation [21]. On this basis, NHEJ pathway can be considered as a promising target to enhance the radiosensitivity of human cancers.

In recent years, remarkable efforts on radiobiology have been directed toward the detection of genes associated with radiosensitivity. Accumulating evidence indicate that many NHEJ-associated genes are disturbed in human cancers and help to predict response to ionizing radiation [25–27]. In the present study, we investigated the influence of XRCC4 on the radiosensitivity of TNBC cells by lentivirus-mediated RNA interference, and found that XRCC4 knockdown sensitized TNBC cells to ionizing radiation. As a core member involved in NHEJ pathway, XRCC4 can interact with ligase IV to repair the DSBs and maintain the stability of the genome [28,29]. XRCC4/DNA ligase IV complex can stimulate the activity of DNA-dependent protein kinase and directly join compatible DNA ends [30]. The deregulation of XRCC4 can lead to malfunction of DNA repair pathway, further causing DNA damage-induced cell death. In line with the previous studies, the results of Comet assay in our study also showed that the DNA damage level was increased in the XRCC4 knockdown group, indicating there was a significant repair delay in the XRCC4-deleted cells. Bertolini et al. [12] indicated that transient depletion of Ku70 and XRCC4 by RNAi could manipulate the NHEJ pathway and sensitize cancer cells to ionizing radiation. Previous studies have provided sufficient evidence that XRCC4 decrease plays a radiosensitization effect in various human cancers [13,14,31]. Our study also suggested that low expression of XRCC4 was a significant predictor of radiosensitivity in patients with TNBC.

Radiotherapy is an important locoregional treatment for TNBC patients. The effects of radiotherapy on cancer control have been shown to be limited to the radioresistance and side effects. In general, radiation dose must be strong enough to ensure the elimination of cancer cells because low-dose palliative treatment may cause poor treatment effect. However, high doses can cause the development of side effects, such as fatigue [32], skin irritation [33], breast swelling and the risk of cardiac disease [34,35]. The severity of side effects in radiation treatment is dose dependent [8]. Thus, the central challenge of radiotherapy is to maximize cancer cell killing and minimize side effects. In addition, the response of individuals to radiation therapy is different [36–38]. Patients with high radiosensitivity could be cured by modest doses of radiation. The heterogeneity of responses to ionizing radiation in patients with TNBC demands a significantly various dose of radiation to achieve a radical cure. This drives the investigators focus to distinguish the radiosensitivity of particular patients to give optimizing radiation dose. Given the emerging view that XRCC4 is involved in the radiosensitivity of TNBC cells and is expressed variously in different individuals, we were tempted to provide a customized dose reduction for TNBC patients with XRCC4 low expression to alleviate the unexpected side effects. In future, patients with TNBC should be encouraged to detect XRCC4 expression prior to radiation treatment, enabling radiologists to identify hypersensitive patients to improve outcome.

## Conclusion

Our report indicated that XRCC4 knockdown by shRNA enhanced the response of TNBC cells to ionizing radiation. Moreover, XRCC4 is expressed variously in patients with TNBC, and can be considered as a prognostic factor for TNBC patients with radiation treatment. Taking together, targeting XRCC4 could contribute to the prediction of radiosensitivity of specific patients and the sensitization of tumor cells. This new therapeutic strategy may promise to improve the outcome of radiotherapy beyond the precise and specific radiation dose to TNBC patients.

## Supporting information

**Supplementary Figure S1:** 

## Abbreviations

DSB: DNA double-strand break
HR: homologous recombination
NHEJ: non-homologous end joining
PFS: progression-free survival
TNBC: triple-negative breast cancer
XRCC4: X-ray repair cross-complementing gene 4
