# Supplementary figures and images for: Silencing of XRCC4 increases radiosensitivity of triple-negative breast cancer cells
Source: Biosci Rep. 2019 Mar 19;39(3):BSR20180893. doi: 10.1042/BSR20180893 (PMC6423307; doi:10.1042/BSR20180893)

**A**

ER

PR

HER-2

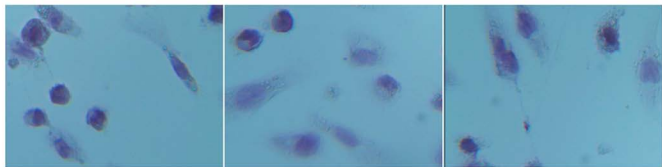**B**

Light

GFP

Merge

NT

Vector

shRNA

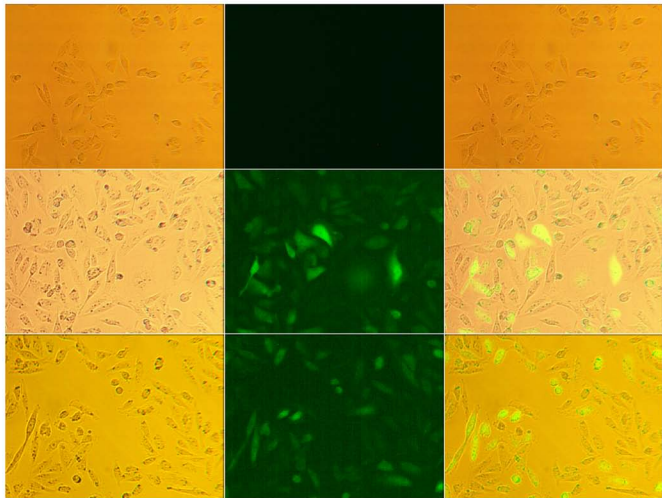

Supplement: Supplementary file 1 [file bsr-39-bsr20180893_Supp1.pdf]
